# Transcriptomic heterogeneity of driver gene mutations reveals novel mutual exclusivity and improves exploration of functional associations

**DOI:** 10.1002/cam4.4039

**Published:** 2021-06-02

**Authors:** Yujia Lan, Wei Liu, Wanmei Zhang, Jing Hu, Xiaojing Zhu, Linyun Wan, Suru A, Yanyan Ping, Yun Xiao

**Affiliations:** ^1^ College of Bioinformatics Science and Technology Harbin Medical University Harbin China

**Keywords:** mutations, functional associations, mutual exclusivity, non small cell lung cancer, cancer genetics

## Abstract

**Background:**

Lung adenocarcinoma (LUAD), as the most common subtype of lung cancer, is the leading cause of cancer deaths in the world. The accumulation of driver gene mutations enables cancer cells to gradually acquire growth advantage. Therefore, it is important to understand the functions and interactions of driver gene mutations in cancer progression.

**Methods:**

We obtained gene mutation data and gene expression profile of 506 LUAD tumors from The Cancer Genome Atlas (TCGA). The subtypes of tumors with driver gene mutations were identified by consensus cluster analysis.

**Results:**

We found 21 significantly mutually exclusive pairs consisting of 20 genes among 506 LUAD patients. Because of the increased transcriptomic heterogeneity of mutations, we identified subtypes among tumors with non‐silent mutations in driver genes. There were 494 mutually exclusive pairs found among driver gene mutations within different subtypes. Furthermore, we identified functions of mutually exclusive pairs based on the hypothesis of functional redundancy of mutual exclusivity. These mutually exclusive pairs were significantly enriched in nuclear division and humoral immune response, which played crucial roles in cancer initiation and progression. We also found 79 mutually exclusive triples among subtypes of tumors with driver gene mutations, which were key roles in cell motility and cellular chemical homeostasis. In addition, two mutually exclusive triples and one mutually exclusive triple were associated with the overall survival and disease‐specific survival of LUAD patients, respectively.

**Conclusions:**

We revealed novel mutual exclusivity and generated a comprehensive functional landscape of driver gene mutations, which could offer a new perspective to understand the mechanisms of cancer development and identify potential biomarkers for LUAD therapy.

## Graphical abstract

We revealed novel mutual exclusivity and generated a comprehensive functional landscape of driver gene mutations, which could offer a new perspective to understand the mechanisms of cancer development and identify biomarkers for LUAD therapy.

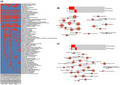

## 1 INTRODUCTION

Lung cancer is a major public health problem all over the world and the second leading cause of death in the world.^1^, ^2^ Lung adenocarcinoma (LUAD) is the most common histologic type of primary lung cancer, accounting for about 40% of all lung cancers.^3^ Its incidence rate is increasing in people who have never smoked, or are aged 20–46 years.^4^ LUAD patients can be divided into five groups using the TNM classifications system.^5^, ^6^ The higher the grade is, the more malignant the tumor is. The standard treatment of LUAD patients is surgery, radiation therapy, chemotherapy, and targeted therapies.^7^, ^8^, ^9^

Some studies have shown that solid tumors contain hundreds or thousands of genetic alterations across multiple cancers, including lung adenocarcinoma.^10^, ^11^, ^12^, ^13^ The vast majority of them are point mutations, which are composed of driver mutations and passenger mutations. The former confer selective growth advantages to tumor cells,^12^, ^14^, ^15^, ^16^ while the latter occur during a large number of cell divisions.^17^, ^18^ The driver gene is defined as one whose mutations increase cell growth under the specific conditions in cells.^19^ There are some driver gene mutations found in LUAD, for example, driver gene EGFR.^20^, ^21^ Targeted therapies against several oncogenic drivers, such as *EGFR* and *BRAF* mutations, have been approved for the precision treatment of LUAD.^22^, ^23^, ^24^ However, patients with the same driver genetic alterations showed extensive genetic and transcriptomic heterogeneity.^25^, ^26^

The heterogeneity of tumors could be a major obstacle for anticancer treatment, which can occur at genetic, transcriptomic, and histological levels. Transcriptomic heterogeneity is very important in cancer researches because mRNAs can be treated as a bridge that links genetic variations and physiological traits.^27^, ^28^ Nowadays, gene expression‐based molecular subtyping has been used in cancers to aid treatment decisions due to the existence of transcriptomic heterogeneity. For example, breast cancer patients can be divided into four intrinsic molecular subtypes (Basal‐like, HER‐2 positive, Luminal A, and Luminal B), which has been proven to be clinically effective.^29^ However, the degree of transcriptomic heterogeneity of LUAD tumors with different driver gene mutations is still unclear, which may be the key to understand the functions of driver gene mutations and improve the therapeutic efficacy in cancers.

The accumulating genetic alterations in cancers do not occur at random, but mutually depend on each other.^30^ Some co‐occurring events were observed in multiple cancers by high‐throughput sequencing data.^31^ For example, driver gene *CTNNB1* and *PIK3CA* cooperatively promote tumor metastasis.^32^ Mutual exclusivity refers to the phenomenon that genetic alterations of genes do not tend to occur in the same sample, which has been widely observed in numerous cancer cohorts.^33^, ^34^ Some well‐known cancer driver genes are mutual exclusivity. The mutually exclusive events, including *BRAF* and *KRAS* mutations (two members of MAPK–ERK pathway), are mutual exclusivity and undergo genetic alterations in lung cancer patients.^35^, ^36^ Similarly, *KRAS* and *EGFR* mutations are also mutually exclusive in LUAD.^37^ What's more, the co‐occurring or mutually exclusive events have been reported to be clinically relevant. For example, the mutual exclusivity of *ATM* and *TP53* mutations in mantle cell lymphoma patients was associated with significantly reduced overall survival.^38^ Thus, it is necessary to comprehensively identify and analyze co‐occurring or mutually exclusive events of mutations to enhance the understanding of tumorigenesis and improve the treatment strategies for precision medicine.

In the present study, we comprehensively characterized the functions of 178 driver gene mutations within specific subtypes across 506 LUAD patients from The Cancer Genome Atlas (TCGA) project. The tumors with driver gene mutations were divided into diverse subtypes based on transcriptomic heterogeneity. Interestingly, we found more co‐occurring and mutually exclusive pairs of subtypes of tumors with driver gene mutations. Furthermore, these mutually exclusive pairs exhibited crucial roles in cancers, including nuclear division, humoral immune response, cell motility, cell differentiation, and blood circulation. Finally, we observed functional and prognostic mutually exclusive triples.

## 2 MATERIALS AND METHODS

### 2.1 Data source

In this study, the mutation profile, gene expression profile, and corresponding clinical metadata (including clinicopathological factors, overall survival [OS], disease‐specific survival [DSS]) of LUAD patients were accessed through The Cancer Genome Atlas (TCGA) portal (https://portal.gdc.cancer.gov). Non‐silent mutations are of great significance for the functional analysis of mutated genes,^39^ so subsequent analysis focused on non‐silent mutations. For the mutation profile, non‐silent somatic mutations (missense mutation, nonsense mutation, translation start site, in‐frame deletion, in‐frame insert, frame‐shift deletion, frame‐shift insert, splice site, and nonstop mutation) remained according to UCSC Genome Browser (http://genome.ucsc.edu).^40^ The data were filtered to exclude patients without mutation data or clinical information. There were 506 LUAD patients having mutation data and gene expression data. A total of 497 patients have available overall survival data and 464 patients have disease‐specific survival data. Besides, the cancer driver genes (*n* = 846) were obtained from public sources in our research. The Catalogue Of Somatic Mutations In Cancer (COSMIC) gene census manually collected more than 700 cancer genes that were mutated and causally implicated in cancer development from literatures.^41^ Matthew H. Bailey et al. used 26 computational tools to identify 299 driver genes in multiple cancers.^42^ The Cancer Genome Atlas Research Network identified 18 significant mutated genes among 412 lung adenocarcinomas.^43^ And, Joshua D Campbell et al. identified 66 driver genes in lung cancers.^44^

### 2.2 Identifying subtypes of tumors with mutations in each driver gene

For each driver gene, we classified the tumor samples harboring mutations in this driver gene into subtypes in three steps. First, the top 2000 most variant genes were selected according to the value of median absolute deviation (MAD) across tumors. Expression values of the above variable genes were log2 transformed and then median‐centered across samples for each gene. Second, we performed consensus clustering to divide the driver gene‐mutated tumors into subtypes by ConsensusClusterPlus R package.^45^ We used Partitioning Around Medoids (PAM) algorithm to implement the unsupervised consensus clustering the Pearson's correlation coefficient as a similarity measure based on the expression data of most variable genes. The optimal number of subtypes was assessed based on 80% sample resampling over 1000 iterations. For driver genes mutations that appeared in more than 20 samples, the optimal number was determined by consensus membership heatmap. For driver gene mutations that mutated in no more than 20 samples, they were divided directly into two subtypes. Third, we calculated the silhouette width of each tumor sample within the same subtypes based on the Pearson distance. Samples with non‐positive silhouette width were regarded as unstable and removed. Subtypes with samples less than five were also removed.

### 2.3 Identifying the functions of mutually exclusive pairs

We selected 17,887 genes which showed detectable expression (counts >1 in at least 30% of LUAD samples). At first, differential expression analysis was performed based on the gene expression profile of LUAD patients by the “DESeq2” R package. Genes with the cutoff criteria of |log2‐fold change| ≥1 and FDR <0.05 between some subtype of tumors with driver gene mutations and wild‐type (WT) patients were regarded as differentially expressed genes (DEGs). Functional enrichment in Gene Ontology (GO) biological processes of the above DEGs was performed using g: profiler,^46^, ^47^ setting a threshold of 0.05 for statistical significance. At this point, the functions of driver gene mutations within subtypes were found.

Then, we identified the functions of mutually exclusive pairs of driver gene mutations within subtypes, which were functional intersections between two subtypes of driver gene mutations of some mutually exclusive pair. Furthermore, the functions of mutually exclusive triples were functional intersection in at least two mutually exclusive pairs.

Visualization of GO enrichment was performed using the EnrichmentMap plugin^48^ in Cytoscape.^49^ Similar GO terms were clustered together based on the similarity between each other using the overlap coefficient. Clusters were manually circled and labeled to highlight the prevalent biological functions among related GO terms.

### 2.4 Survival analyses

For survival analysis, overall survival and disease‐specific survival were used as the end points. The Kaplan–Meier method was performed for visualization purposes and the differences between survival curves were calculated by log‐rank test. Univariate and multivariate Cox proportional hazards regression models were applied to estimate the prognostic capability of mutually exclusive triples. The *p* values smaller than 0.05 were considered to be statistically significant. All of the statistical analyses were performed using R software (www.r‐project.org).

## 3 RESULTS

### 3.1 The mutually exclusive/co‐occurring events of driver gene mutations across LUAD patients

**FIGURE 1:**
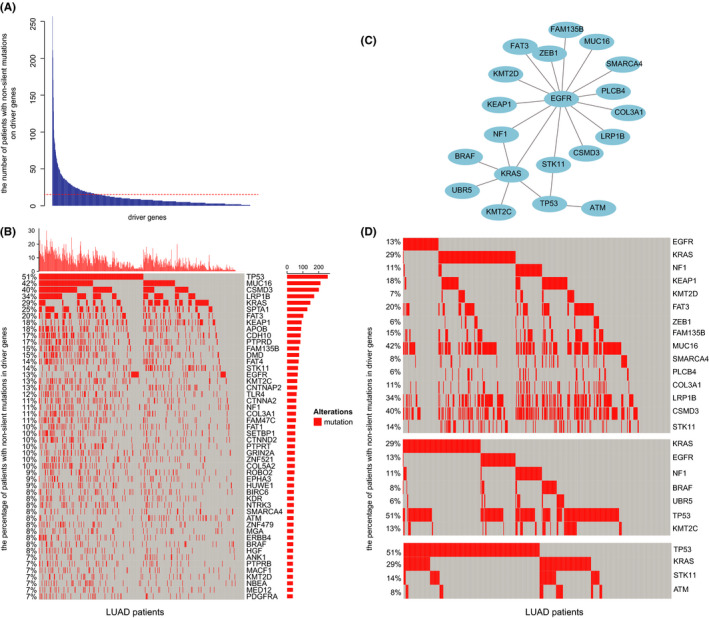
The mutually exclusive events of driver gene mutations across LUAD patients. (A) The numbers of LUAD patients with non‐silent mutations in driver genes. The red line indicated 15 patients. (B) Heatmap of the top 50 frequently mutated driver genes. The red square represented the mutations of driver genes. The X‐axis means LUAD patients and the Y‐axis means the percentage of patients with non‐silent mutations in driver genes. (C) All 21 mutually exclusive events were identified in 178 driver gene mutations, including 20 driver genes. (D) The mutual exclusivity of *EGFR* mutations, *KRAS* mutations, or *TP53* mutations. Each column represents mutated tumors. The X‐axis means LUAD patients and the Y‐axis means the percentage of patients with non‐silent mutations in driver genes

Driver gene mutations promote tumorigenesis and play major impacts on patient outcome.^50^, ^51^ To explore the functions and associations among driver gene mutations in LUAD, we obtained 846 driver genes from public sources and mutation profile of patients from TCGA. Among the 506 LUAD patients, we identified a total of 11,519 non‐silent mutations in driver genes. The non‐silent mutations consisted of 9509 missense mutations (83%), 1003 nonsense mutations (9%), 403 splice site mutations (3%), 9 translation start site (0.08%), 2 nonstop mutation (0.02%), 514 frameshift indels (4%), and 79 in‐frame indels (0.7%). There were 783 driver genes with at least one mutation among LUAD patients. And, 178 driver genes were mutated in more than 15 patients (> 3%) among the above genes^51^, ^52^, ^53^ (Figure 1A), which were used for subsequent analysis. Seven of these genes appeared in at least 100 patients, including driver genes *TP53* (*n* = 257), *MUC16* (*n* = 211), *CSMD3* (*n* = 201), LRP1B (*n* = 171), *KRAS* (*n* = 147), *SPTA1* (*n* = 128), and *FAT3* (*n* = 103). These seven driver genes could be verified by the oncoKB database^54^ and by other researches.^55^, ^56^, ^57^, ^58^

In the profile of non‐silent mutations in driver genes, which presented in at least 7% of LUAD patients, mutually exclusive or co‐occurring phenomena were observed (Figure 1B). Therefore, 21 mutually exclusive events were significantly identified in LUAD using the DISCOVER method,^59^ involving 20 driver gene mutations (*q* value <0.05, Figure 1C). We observed that *EGFR* mutations were mutually exclusive with 14 driver gene mutations, such as driver oncogene *KRAS* and *KMT2D* mutations (Figure 1D). *KRAS* mutations and six driver gene mutations were mutually exclusive (Figure 1D). In addition, oncogene *TP53* mutations were not only mutually exclusive with *KRAS* mutations, but also with *STK11* and *ATM* mutations (Figure 1D). However, no significantly co‐occurring events were found in our research. These results pointed to the existence of mutually exclusive driver gene mutations across LUAD patients.

### 3.2 High transcriptomic heterogeneity of tumors with driver gene mutations

**FIGURE 2:**
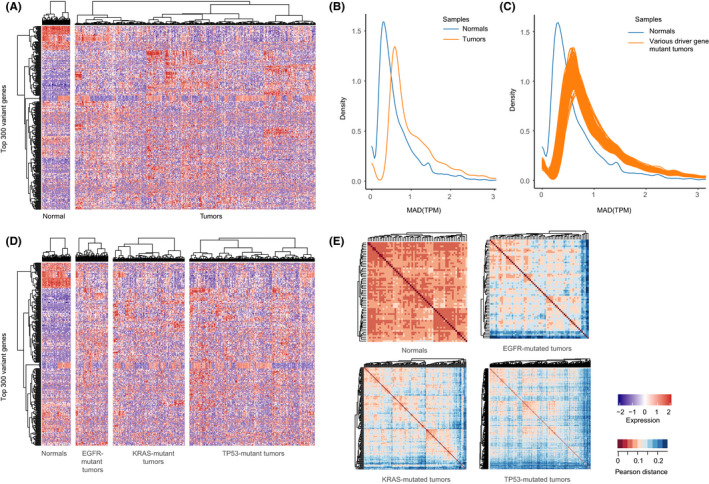
High transcriptomic heterogeneity in tumors with driver gene mutations. (A) A heatmap showed the unsupervised hierarchical clustering of LUAD tumors (*n* = 506) and normal samples (*n* = 59) by the most variable genes (*n* = 300). (B) The MAD distribution of all genes across all tumor and normal samples. The blue and orange lines representing tumor and normal samples, respectively. (C) The MAD distribution of all genes across samples with driver gene mutations. The blue and orange lines representing gene‐mutated tumors and normal samples, respectively. (D) A heatmap was shown by unsupervised hierarchical clustering of the most variable genes (*n* = 300) among *EGFR*‐, *KRAS*‐, and *TP53*‐mutated tumors and normal samples. (E) Pairwise comparison of transcriptomic profiles in normal samples and *EGFR*, *KRAS*, and *TP53* mutant tumors. The heatmaps were plotted using unsupervised hierarchical clustering with Pearson distance (1‐Pearson correlation coefficient)

We sought to examine the extent of the transcriptomic heterogeneity of 506 LUAD tumors and 59 adjacent normal samples. Genes exhibited stable high or low expression in normal samples, while the expression of genes was quite messy in tumors through unsupervised cluster analysis on the top 300 variant genes. This phenomenon indicated that transcriptomic heterogeneity increased in tumors (Figure 2A). To quantificationally estimate the transcriptomic heterogeneity for samples, we calculated the median absolute deviation of all genes in tumor samples. The MAD values of tumors were significantly higher than the normal samples (*p* < 0.001, Wilcoxon rank sum paired test, Figure 2B).

Tumors with various driver gene mutations also consistently had higher transcriptomic heterogeneity compared with the normal samples (*p* < 0.001, Wilcoxon rank‐sum paired test, Figure 2C). Besides, cluster heatmap showed that genes expressed inconsistently in their respective tumor samples with driver gene mutations, such as *EGFR*‐, *KRAS*‐, and *TP53*‐mutated tumors (Figure 2D). Meanwhile, we compared the transcriptomic similarity within tumors with driver gene mutations and normal samples by calculating the Pearson distance. A tight correlation among normal samples was observed (Figure 2E). In contrast, tumors with various driver gene mutations showed great diversity, for example, *EGFR*‐mutated tumors, *KRAS*‐mutated tumors, and *TP53*‐mutated tumors (Figure 2E). This phenomenon showed that unknown homogeneous clusters were hidden in the driver gene‐mutated tumors. These results implied that tumors with driver gene mutations were needed to be divided into diverse subtypes.

### 3.3 Identification of subtypes of tumors with driver gene mutations

**FIGURE 3:**
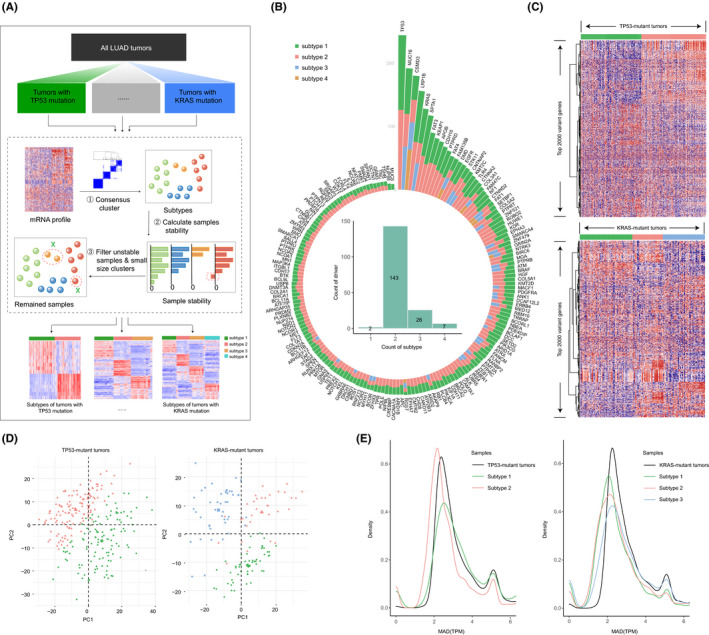
Subtypes of tumors with driver gene mutations. (A) The overview of identification of subtypes of tumors with driver gene mutations. (B) Ring bar plots (outer) showing the count of subtypes and samples of each driver gene. Bar plots (inner) showing the count of driver genes divided into various subtypes. (C) Gene expression heatmap (red =high expression; blue =low expression) of *TP53*‐ (*n* = 244) and *KRAS* (*n* = 131)‐mutated tumors by the 2000 most variable genes. (D) A principal component analysis (PCA) of *TP53*‐ and *KRAS*‐mutated tumors. (E) The MAD distribution of the most variable genes in *TP53* (left) and *KRAS* (right) mutant tumors and corresponding subtypes

Next, we identified molecular subtypes of LUAD tumors in three steps (Figure 3A). First, LUAD tumors were assigned to 178 groups by driver gene mutations. Then, the consensus clustering algorithm was applied on the transcriptomic profiles of each group of driver gene‐mutated tumors to determine subtypes. In order to retain representative samples in each subtype, we eventually performed silhouette width analysis to exclude the unstable samples. To ensure sufficient statistical power, there were five subtypes (*COL2A1* subtype two, *SMAD4* subtype two, *IL7R* subtype two, *MUC6* subtype one, and *NUP98* subtype two) with the small sample size (*n* < 5) excluded in our study. We finally divided the tumors with 178 driver gene mutations into various subtypes and the majority of driver‐mutated tumor groups had two subtypes (143/178, Figure 3B).

Here, we took the critical driver gene *TP53* and *KRAS* as examples. As shown in the cluster heatmap on top 2000 variable genes, a discriminable expression pattern between two subtypes of *TP53* mutations was observed (Figure 3C top). *KRAS*‐mutated tumors, divided into three subtypes, showed a similar phenomenon (Figure 3C bottom). The clustering robustness was assessed with principal component analysis, *TP53*‐ and *KRAS*‐mutated tumors formed two and three separate groups, respectively, which supported the classification of subtypes (Figure 3D). In order to observe the changes in transcriptomic heterogeneity between tumors with driver gene mutations and their corresponding subtypes, we compared the MAD of top variable genes. Transcriptomic heterogeneity of *TP53* subtype two decreased significantly (*p* < 0.001, Wilcoxon rank‐sum paired test) compared with all tumors with *TP53* mutation, while subtype one changed little (Figure 3E). Besides, all the three subtypes of *KRAS*‐mutated tumor samples decreased significantly (*p* < 0.0001, Wilcoxon rank‐sum paired test, Figure 3E). These results indicated that the subtypes of each driver gene‐mutated tumors were composed of stable subtypes with lower transcriptomic heterogeneity.

For further analysis of functions of driver gene mutations in subtypes, we identified DEGs between the subtypes of tumors with driver gene mutations and wild‐type samples and performed functional enrichment analysis based on these DEGs using g: profiler.^46^, ^47^ We found some key biological functions of driver gene mutations within subtypes. *TP53* subtype one was significantly enriched in cell cycle, cell division, and nuclear division (adjusted enrichment *p* < 0.05, Figure S1A). The cell cycle is associated with a cancer hallmark, self‐sufficiency in growth signals.^60^, ^61^, ^62^ We also identified functions related to other cancer hallmarks. The humoral immune process of *TP53* subtype two was related to tumor‐promoting inflammation and evading immune detection (Figure S1A).^60^, ^61^, ^62^ Importantly, *KRAS* subtype three was specifically enriched in the immune system process, such as lymphocyte proliferation, leukocyte proliferation, and T cell proliferation (Figure S1B). These results suggested that different subtypes of the same driver gene mutations in LUAD patients had diverse functions.

### 3.4 The co‐occurring or mutually exclusive pairs of driver gene mutations within subtypes

**FIGURE 4:**
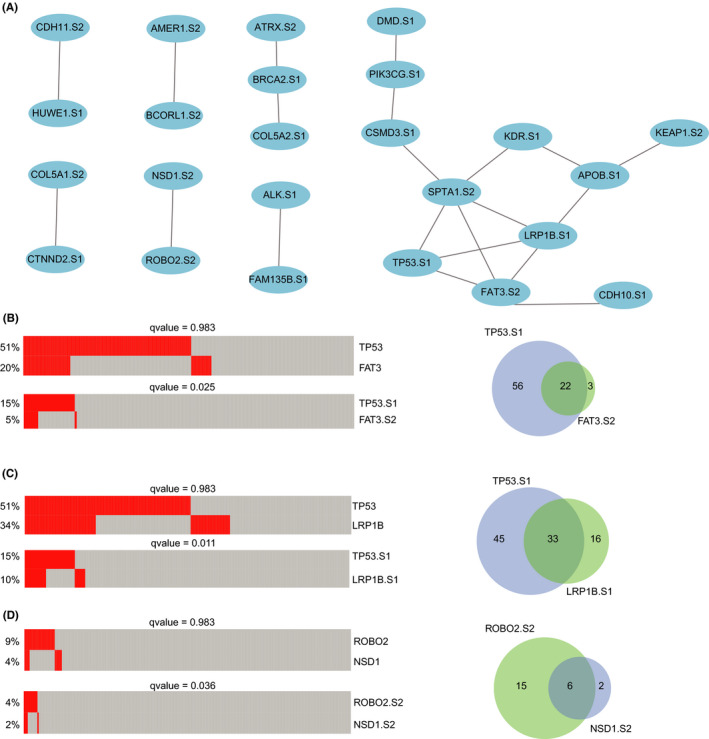
The co‐occurring pairs of driver gene mutations within subtypes. (A) Twenty‐one co‐occurring pairs of 20 driver gene mutations within subtypes. S1, S2, and S3 mean subtype one, subtype two, and subtype three, respectively. (B–D) The co‐occurring pairs of *TP53* subtype one–*FAT3* subtype two, *TP53* subtype one–*LRP1B* subtype one, *ROBO2* subtype two–*NSD1* subtype two, respectively. The heatmap on the left showed the mutational status of driver gene mutations within subtypes (red for mutation) for each sample (each column). Venn diagrams on the right showing the overlap of samples with co‐occurring pairs

We wanted to explore whether the co‐occurring or mutually exclusive pairs existed in driver gene mutations within subtypes. At first, we identified 21 significantly co‐occurring pairs among 394 subtypes of 178 driver gene mutations using the DISCOVER method, including 24 subtypes and 24 driver genes (*q* value <0.05, Figure 4A). Among these co‐occurring pairs, we found *TP53* subtype one was co‐occurring with *FAT3* subtype two (*q* value =0.025, Figure 4B). There were 22 common samples shared by these two subtypes, encompassing 78 samples of *TP53* subtype one and 25 samples of *FAT3* subtype two. However, driver gene *TP53* mutations were not co‐occurring with *FAT3* mutations (*q* value =0.983). Another example of *TP53* subtype one and *LRP1B* subtype one was observed, which was identified as a co‐occurring pair (*q* value =0.011, Figure 4C). This co‐occurring pair was commonly detected in 33 samples (54 samples of *LRP1B* subtype one). A similar phenomenon was also observed between *ROBO2* subtype two and *NSD1* subtype two (*q* value =0.036, Figure 4D). These results revealed that more co‐occurring pairs of subtypes of tumors with driver gene mutations were found than tumors with mutations in driver genes.

**FIGURE 5:**
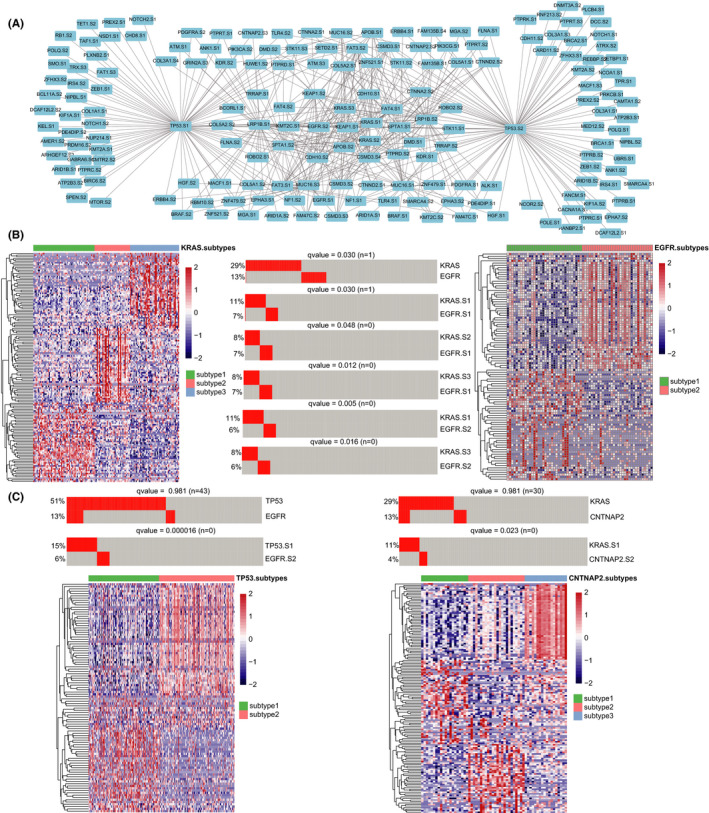
The mutually exclusive pairs of driver gene mutations within subtypes. (A) A total of 494 mutually exclusive pairs of 112 driver gene mutations within subtypes. S1, S2, and S3 mean subtype one, subtype two, and subtype three, respectively. (B) The mutually exclusive pair of subtypes of *KRAS* mutations and *EGFR* mutations (middle). The heatmap showed the top 50 differentially expressed genes between subtypes of *KRAS*‐mutated (left) and *TP53*‐mutated (right) tumors. (C) The mutually exclusive pairs of *TP53* subtype one–*EGFR* subtype two and *KRAS* subtype one–*CNTNAP2* subtype two

We also identified 494 mutually exclusive pairs of driver gene mutations within subtypes (Figure 5A), including 179 subtypes of 112 driver gene mutations. There was an interesting phenomenon that up to 168 subtypes were mutually exclusive with two subtypes of *TP53*. *TP53* subtype one and *TP53* subtype two were mutually exclusive with 82 subtypes and 91 subtypes of driver gene mutations, respectively. Only five subtypes (*SETD2* subtype one, *KEAP1* subtype one, *KRAS* subtype two, *ATM* subtype three, and *KRAS* subtype three) were mutually exclusive with both two subtypes of *TP53* mutations (Table S1). Most subtypes were mutually exclusive with one of the *TP53* mutations’ subtypes. Although *PIK3CA* subtype two was mutually exclusive with *TP53* subtype one, it was not mutually exclusive with *TP53* subtype two. The same result was observed between *TP53* subtype two and *PTPRT* subtype two (Figure S2).

Among 494 mutually exclusive pairs, some pairs were not only identified among subtypes of tumors with driver gene mutations but also among driver gene mutations. Let us take an example of the driver gene *KRAS* and *EGFR*. *KRAS* mutations and *EGFR* mutations were significantly mutually exclusive events (*q* value =0.030, Figure 5B). At the subtype level, tumors with *KRAS* mutations and *EGFR* mutations were divided into three and two groups, respectively. Except for the pair of KRAS subtype two and *EGFR* subtype two, the other five pairs of subtypes were all significantly mutually exclusive (*q* value <0.05). Surprisingly, most of the mutually exclusive pairs were only observed at the subtype level. *TP53* subtype one was mutually exclusive with *EGFR* subtype two (*q* value =0.000016), where tumors with *TP53* mutations were divided into two groups (Figure 5C). However, *TP53* mutations were not significantly mutually exclusive with *EGFR* mutations (*q* value =0.981). A similar phenomenon was found in the pair of *KRAS* subtype one and *CNTNAP2* subtype two (Figure 5C). These results revealed that more mutually exclusive or co‐occurring pairs were identified among subtypes of tumors with driver gene mutations based on transcriptomic heterogeneity.

### 3.5 Identification of biological functions of driver gene mutations within subtypes

**FIGURE 6:**
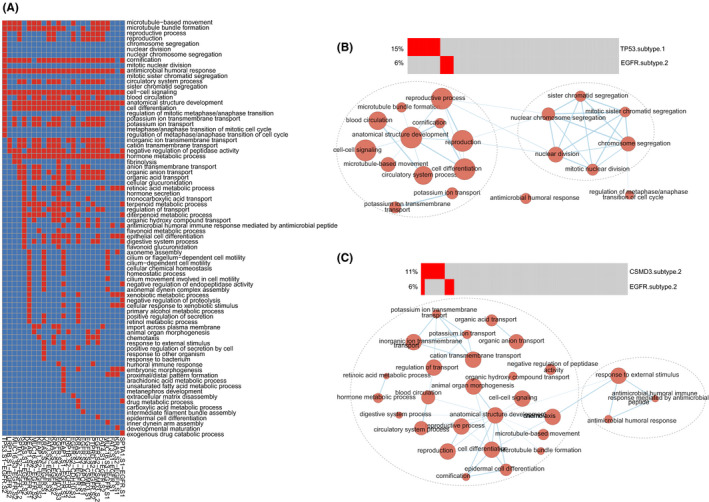
The functions of mutually exclusive pairs of *EGFR* mutations within subtypes. (A) The heatmap showed the functions of mutually exclusive pairs of *EGFR* mutations within subtypes. Red represented some mutually exclusive pair was significantly enriched in one function. (B–C) The functions of mutually exclusive pairs of *TP53* subtype one–*EGFR* subtype two and *CSMD3* subtype two–*EGFR* subtype two. Functional enrichment results were visualized using EnrichmentMap plugin in Cytoscape. Node size was proportional to the size of the functional gene set. Clusters were manually circled

Subsequently, we identified the functions of mutually exclusive pairs among driver gene mutations within subtypes by functional enrichment analysis (see Methods). As a result, numerous biological processes were significantly enriched for mutually exclusive pairs, such as cell cycle, cell adhesion, and cell differentiation. There were 78 important biological functions found among two subtypes of *EGFR* mutations and their mutually exclusive events, including 25 subtypes of 13 driver gene mutations (Figure 6A). The mutually exclusive pair (*TP53* subtype one and *EGFR* subtype two) was specifically enriched in nuclear chromosome segregation and nuclear division (Figure 6B). Besides, the humoral immune response was found in the mutually exclusive events (*CSMD3* subtype two and *EGFR* subtype two) (Figure 6C), which was useful for autoimmunogenic human tumor antigens and cancers.^63^, ^64^ The similar results were observed in other subtypes of driver gene mutations. Take *KRAS* mutations as an example, the pair of *KRAS* subtype three and *TP53* subtype one was associated with cell motility, cell differentiation, and blood circulation (Figure S3). These functions played key roles in cancer progression.^65^, ^66^, ^67^ In addition, *TP53* mutations and *RB1* mutations were synthetic lethality in the SynLethDB^68^ database. The common presence of the two driver genes could kill cells. All findings suggested that we could identify crucial biological processes of driver gene mutations according to the analysis of mutual exclusion of subtypes of driver gene‐mutated tumors.

### 3.6 The functions of mutually exclusive triples

**FIGURE 7:**
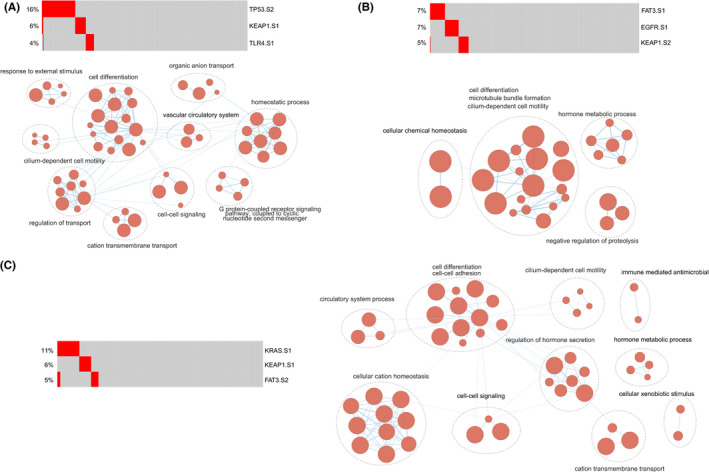
The functions of mutually exclusive triples. (A–C) The functions of mutually exclusive triples of *TP53* subtype two–*KEAP1* subtype one–*TLR4* subtype one, *KEAP1* subtype two–*FAT3* subtype one–*EGFR* subtype one, and *FAT3* subtype two–*KRAS* subtype one–*KEAP1* subtype one. Functional enrichment results were visualized using EnrichmentMap plugin in Cytoscape. Node size was proportional to the size of the functional gene set. Clusters were manually circled and labeled

Furthermore, we identified 79 mutually exclusive triples among driver gene mutations within subtypes (Table S2). In the mutually exclusive triple, every two subtypes were mutually exclusive. There were a number of important biological processes found related to these mutually exclusive triples. Among these mutually exclusive triples, the triple (*TP53* subtype two–*KEAP1* subtype one–*TLR4* subtype one) harbored the most functions (*n* = 70), including cell differentiation, homeostatic process, vascular circulatory system, cell–cell signaling, and cilium‐dependent cell motility (Figure 7A). We found 38 mutually exclusive triples were significantly enriched in cell motility, which were associated with cancer invasion and metastasis.^69^, ^70^ For example, the functions of a mutually exclusive triple (*KEAP1* subtype two–*FAT3* subtype one–*EGFR* subtype one) were cilium‐dependent cell motility, cell differentiation, microtubule bundle formation, and cellular chemical homeostasis (Figure 7B). The triple, *FAT3* subtype two–*KRAS* subtype one–*KEAP1* subtype one, was significantly enriched in cell–cell adhesion, circulatory system process, and immune‐mediated antimicrobial (Figure 7C).

### 3.7 The prognostic value of mutually exclusive triples

**FIGURE 8:**
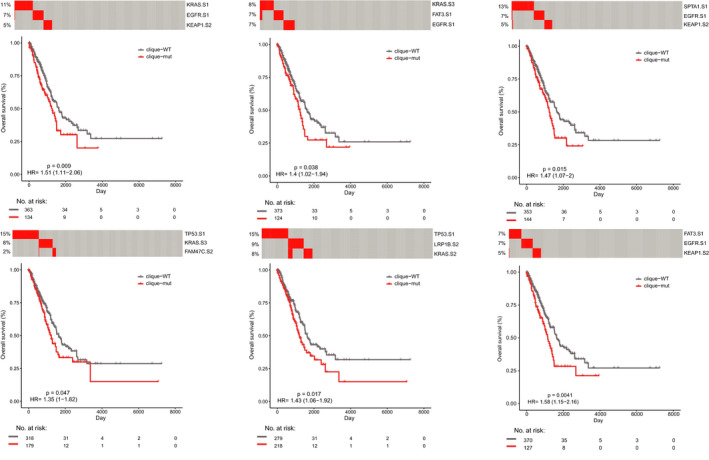
The associations of mutually exclusive triples with clinical outcome. Comparison of overall survival among patients carrying mutually exclusive triples (red line) and cases harboring unmutated genes (gray line) by Kaplan–Meier analysis (with log‐rank *p* values) in the cohort of LUAD patients from TCGA

Finally, we explored the prognostic value of 78 mutually exclusive triples. There were six mutually exclusive triples significantly predicting the overall survival of LUAD patients using the log‐rank test (*p* < 0.05, Figure 8). The samples with these mutually exclusive triples had significantly shorter OS compared with WT sequences (*p* < 0.05). To assess whether the survival prediction ability of the prognostic triples was independent of other clinicopathologic factors in LUAD, univariate and multivariable Cox regression analyses were performed. The covariables included age, gender, AJCC stage, T stage, *N* stage, and these six triples. The patients harboring “*TP53* subtype one–*KRAS* subtype 3–*FAM47C* subtype 2” (HR =1.405, 95% CI 1.014–1.945, *p* = 0.041, Table 1), “*KEAP1* subtype two – *FAT3* subtype one–*EGFR* subtype one” (HR =1.440, 95% CI 1.023–2.027, *p* = 0.036, Table 2) independently predicted poor OS of the patients with LUAD. The other clinical factors, *N* stage, was also independently associated with shorter OS of patients (*p* = 0.019 and *p* = 0.013, respectively).

**TABLE 1:** Multivariate analysis for a mutually exclusive triple of overall survival in the cohort (TP53 subtype one–KRAS subtype three–FAM47C subtype two)

Variables		Univariate			Multivariate	
	HR	95% CI	*p* value	HR	95% CI	*p* value
Age	1.009	0.994–1.025	0.249	1.012	0.996–1.029	0.140
AJCC stage						
Stage II versus I	2.345	1.624–3.388	<0.001^*^	1.046	0.556–1.968	0.890
Stage III versus I	3.495	2.379–5.133	<0.001^*^	1.59	0.626–4.034	0.329
Stage IV versus I	3.341	1.834–6.088	<0.001^*^	2.123	0.995–4.531	0.052
T stage						
T2 versus T1	1.409	0.984–2.018	0.061	1.232	0.845–1.795	0.278
T3 versus T1	3.027	1.798–5.097	<0.001^*^	2.534	1.341–4.788	0.004
T4 versus T1	3.117	1.641–5.920	<0.001^*^	1.667	0.778–3.571	0.189
*N* stage						
N1 versus N0	2.363	1.669–3.346	<0.001^*^	2.025	1.124–3.648	0.019^*^
N2 versus N0	3.115	2.123–4.569	<0.001^*^	1.630	0.698–3.085	0.258
N3 versus N0	3.842	0‐Inf	0.994	1.751	0‐Inf	0.994
Gender						
Male versus Female	1.050	0.779–1.416	0.747	0.882	0.641–1.214	0.441
TP53 subtype one–KRAS subtype three–FAM47C subtype two						
Mutations versus wild type	1.345	0.993–1.820	0.055	1.405	1.014–1.945	0.041^*^

Significant *p* values are labeled with *(*p* < 0.05).

**TABLE 2:** Multivariate analysis for a mutually exclusive triple of overall survival in the cohort (KEAP1 subtype two–FAT3 subtype one–EGFR subtype one)

Variables		Univariate			Multivariate	
	HR	95% CI	*p* value	HR	95% CI	*p* value
Age	1.009	0.994–1.025	0.249	1.010	0.994–1.026	0.237
AJCC stage						
Stage II versus I	2.345	1.624–3.388	<0.001*	0.998	0.527–1.890	0.995
Stage III versus I	3.495	2.379–5.133	<0.001*	1.110	0.429–2.877	0.829
Stage IV versus I	3.341	1.834–6.088	<0.001*	1.641	0.746–3.607	0.218
T stage						
T2 versus T1	1.409	0.984–2.018	0.061	1.264	0.866–1.843	0.224
T3 versus T1	3.027	1.798–5.097	<0.001*	2.446	1.299–4.607	0.006*
T4 versus T1	3.117	1.641–5.920	<0.001*	2.062	0.948–4.484	0.068
*N* stage						
N1 versus N0	2.363	1.669–3.346	<0.001*	2.127	1.176–3.846	0.013*
N2 versus N0	3.115	2.123–4.569	<0.001*	2.274	0.975–5.302	0.057
N3 versus N0	3.842	0–Inf	0.994	2.570	0–Inf	0.994
Gender						
Male versus Female	1.05	0.779–1.416	0.747	0.951	0.695–1.302	0.755
KEAP1 subtype two–FAT3 subtype one–EGFR subtype one						
Mutations versus wild type	1.573	1.144–2.163	0.005*	1.440	1.023–2.027	0.036*

Significant *p* values are labeled with *(*p* < 0.05).

In addition, we investigated the associations of the mutually exclusive triples and disease‐specific survival of LUAD patients. We found that eight mutually exclusive triples significantly predicted shorter DSS of patients by log‐rank test (*p* < 0.05, Figure S4). Furthermore, one mutually exclusive triple (*TP53* subtype one–*MUC16* subtype one–*KRAS* subtype three) significantly independently predicted the survival of patients by univariate and multivariable Cox regression analyses (HR = 1.475, 95% CI 0.986–2.207, *p* = 0.048, Table S3), adjusting for other clinicopathologic factors. These results indicated that the predictive ability of two mutually exclusive triples and one mutually exclusive triple was independent of clinicopathological factors for OS and DSS in LUAD, respectively.

## 4 DISCUSSION

In this study, we comprehensively characterized the functions of 178 driver gene mutations within specific subtypes across 506 LUAD patients from TCGA. We identified 21 significantly mutually exclusive events of driver gene mutations. Because of increasing transcriptomic heterogeneity of tumors with driver gene mutations, they were divided into different subtypes. Notably, using mutual exclusivity, more co‐occurring (*n* = 21) and mutually exclusive pairs (*n* = 494) were found among driver gene mutations within subtypes. The observation motivated us to explore the functions of mutually exclusive pairs. These mutually exclusive pairs of mutations within subtypes played crucial roles in cancers, including nuclear chromosome segregation, nuclear division, humoral immune response, cell motility, cell differentiation, and blood circulation. These results indicated that we could identify more crucial and refined functions of driver gene mutations according to the analysis of mutual exclusion of subtypes of driver gene‐mutated tumors. At last, 79 mutually exclusive triples were significantly enriched in cell differentiation, cell motility, cilium‐dependent cell motility, and cellular chemical homeostasis. We also observed three prognostic mutually exclusive triples, which independently predicted the survival of LUAD patients. In summary, we found novel mutual exclusivity and functional associations of driver gene mutations based on transcriptomic heterogeneity, which could offer a new perspective to understand the mechanisms of cancer development.

In recent studies, accumulating evidence supported that the heterogeneity of LUAD tumors plays an important role in tumor progression, and LUAD is composed of subtypes distinguishable only at the molecular level.^71^, ^72^, ^73^ Toshiyuki Takeuchi et al. established a basis for expression profile‐defined classification, which can classify adenocarcinomas into two major types.^74^ It should be noted that even the tumors with the same driver gene mutations can cause different progression.^75^ In this study, we have observed high transcriptomic heterogeneity in LUAD tumor samples with various driver gene mutations. And, most tumor samples with driver gene mutation can be divided into at least two subtypes. Interestingly, subtypes of the same driver gene mutations were enriched in different functions related to cancer progression. This suggested that transcriptomic heterogeneity, as a confounding factor, may hide the various function of driver genes, which can be resolved with subtypes.

In this research, we identified many biological functions of mutually exclusive pairs of driver gene mutations within subtypes, which played crucial roles in tumorigenesis. Some of these functions were associated with cancer hallmarks, including self‐sufficiency in growth signals, tumor‐promoting inflammation, and evading immune detection.^60^, ^61^, ^62^ For example, the mutually exclusive pair, *TP53* subtype one–*EGFR* subtype two, was not only significantly enriched in known functions of driver genes, but also in more refined functions, including sister chromatid segregation, mitotic sister chromatid segregation, and microtubule bundle formation. The famous driver gene *TP53*, a tumor suppressor gene, is the most frequently mutated gene (>50%) in cancer, which plays a crucial role in preventing cancer formation, such as cell cycle and cell differentiation.^76^, ^77^, ^78^, ^79^ *EGFR* mutations are related to a number of important functions in cancers, including LUAD and glioblastoma,^80^, ^81^ which regulate cell–cell adhesion division and cell differentiation.^82^, ^83^ A similar phenomenon was observed in mutually exclusive triples, such as *TP53* subtype two–*KEAP1* subtype one–*TLR4* subtype one. These findings revealed that the functions of driver gene mutations within subtypes were more refined and were associated with cancer hallmarks, which demonstrated the functions we identified were reliable.

In this research, there were three mutually exclusive triples that independently predicted shorter survival of LUAD patients than the wild‐type samples, namely “*TP53* subtype one–*KRAS* subtype three–*FAM47C* subtype two,” “*KEAP1* subtype two–*FAT3* subtype one ‐ *EGFR* subtype one,” and “*TP53* subtype one–*MUC16* subtype one–*KRAS* subtype three.” Although, *TP53* mutations,^84^, ^85^ KRAS mutations,^86^, ^87^ and *KEAP1* mutations^88^ were predictive of survival of lung cancer patients. This result revealed that mutually exclusive triples could serve as biomarkers of LUAD patients or therapeutic targets. However, these two prognostic triples were needed more data to evaluate their effectiveness in cancers.

## CONFLICT OF INTEREST

The authors declare that they have no conflict of interest.

## Supporting information

Fig S1‐S4‐Table S1‐S3

## Data Availability

The data of this study were freely available from public resources. Because this study did not include humans or animals, the ethical approval was not applicable.
