# Supplementary material for: Transcriptomic heterogeneity of driver gene mutations reveals novel mutual exclusivity and improves exploration of functional associations
Source: Cancer Med. 2021 Jun 2;10(14):4977–93. doi: 10.1002/cam4.4039 (PMC8290236; doi:10.1002/cam4.4039)
Supplement: Supplementary file 1 — Fig S1‐S4‐Table S1‐S3 [file CAM4-10-4977-s001.doc]

**SUPPLEMENTARY INFORMATION**

**Transcriptomic heterogeneity of driver gene mutations reveals novel mutual exclusivity and improves exploration of functional associations**

**Supplementary Figures**

**
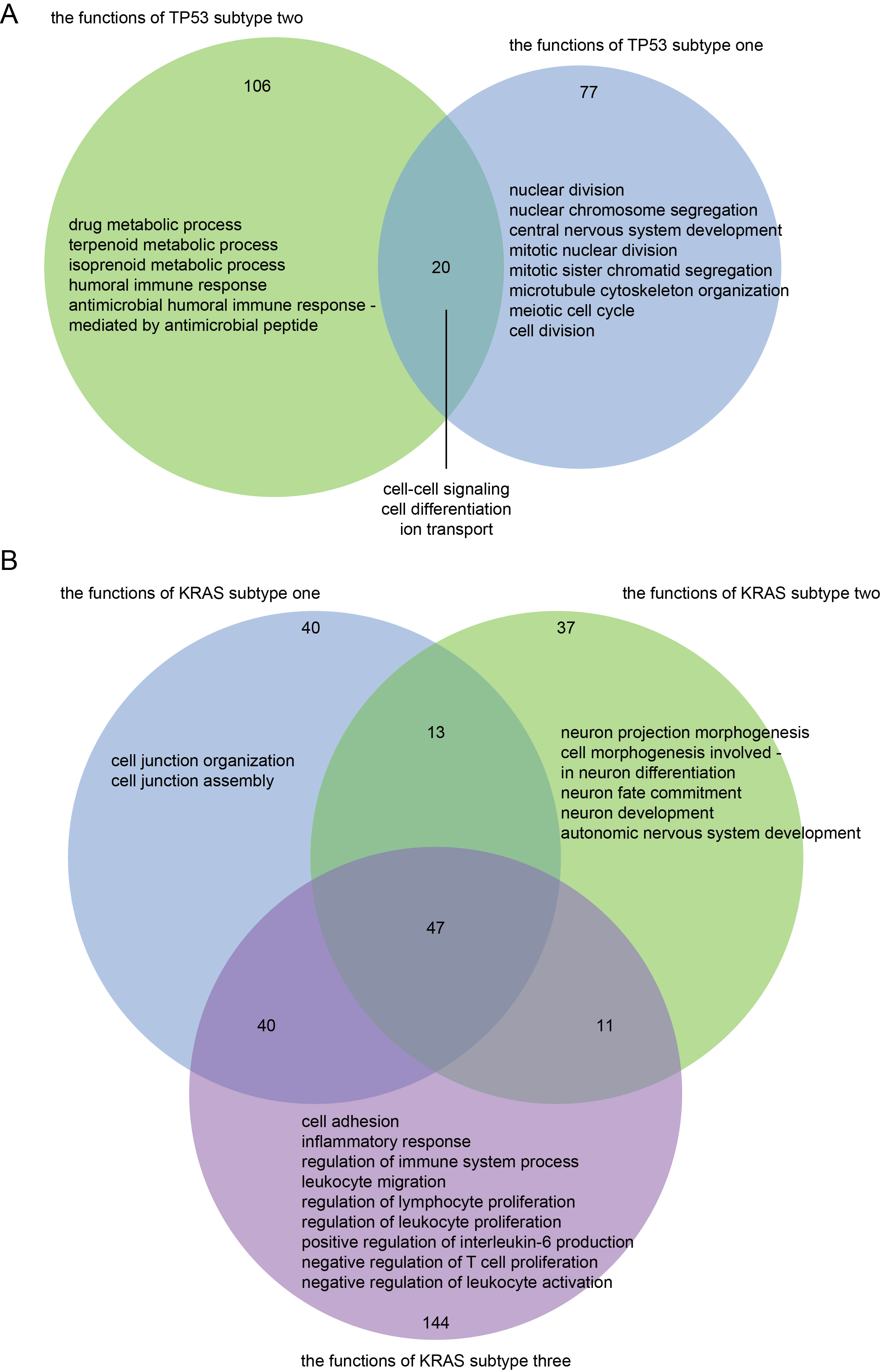
**

**Fig. S1** (A) The functions of TP53 subtype one and TP53 subtype two. Venn diagrams showing the overlap of these two subtypes. (B) The functions of KRAS subtype one, KRAS subtype two and KRAS subtype three.

**
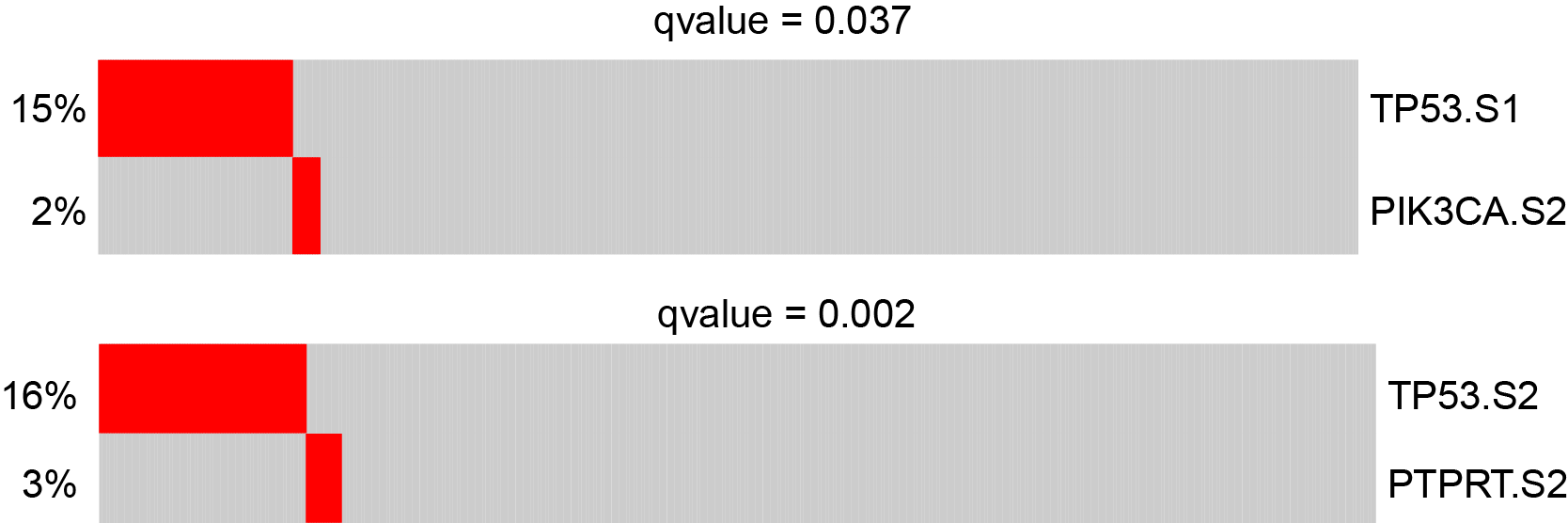
**

**Fig. S2** The mutually exclusive pairs of ‘TP53 subtype one - PIK3CA subtype two’ and ‘TP53 subtype two – PTPRT subtype two’. Each column represents mutated-tumors.

**
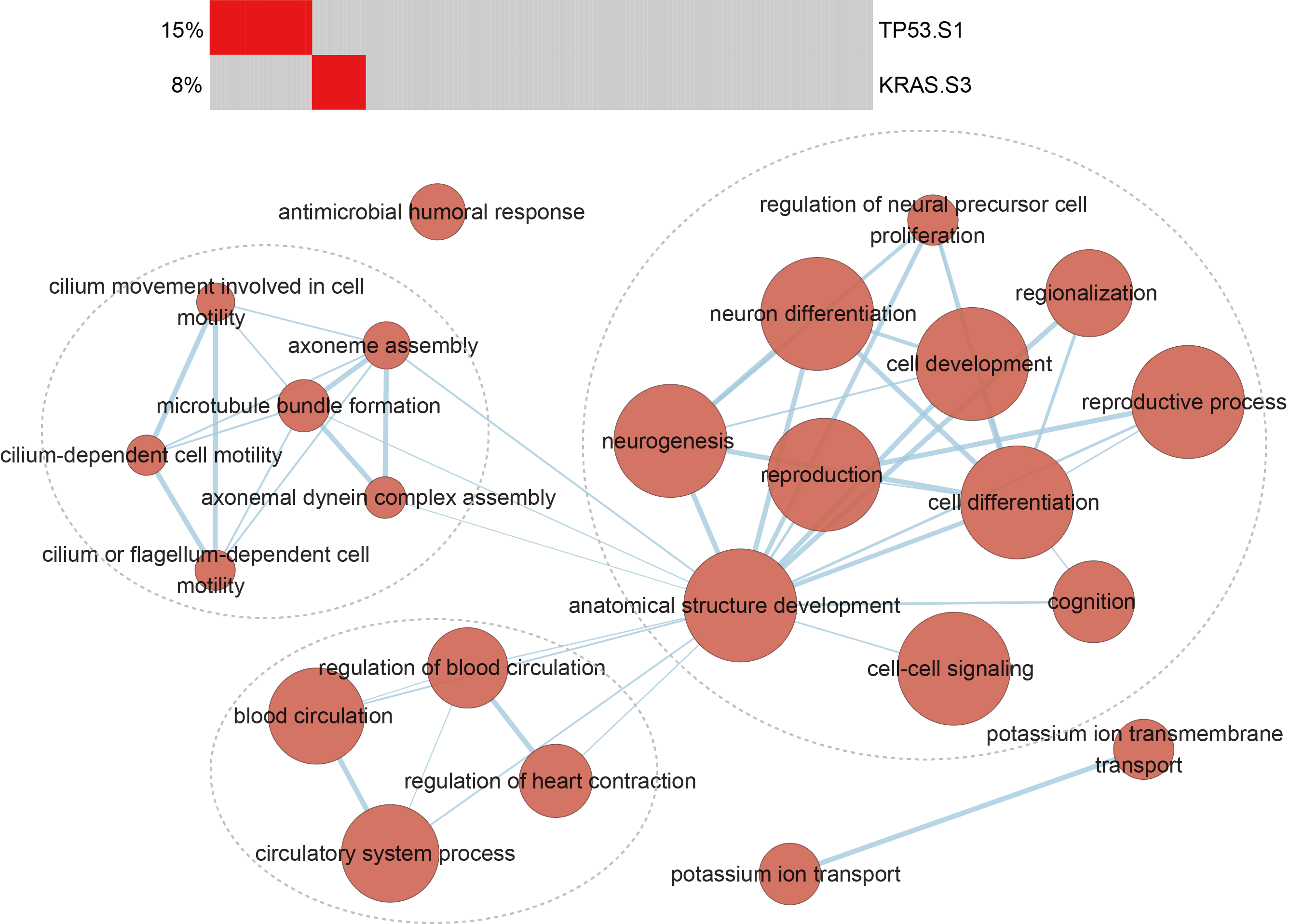
**

**Fig. S3** The functions of the mutually exclusive pair of TP53 subtype one and KRAS subtype three. Functional enrichment results were visualized using Enrichmentmap plugin in Cytoscape. Node size was proportional to the size of the functional gene set. Clusters were manually circled.


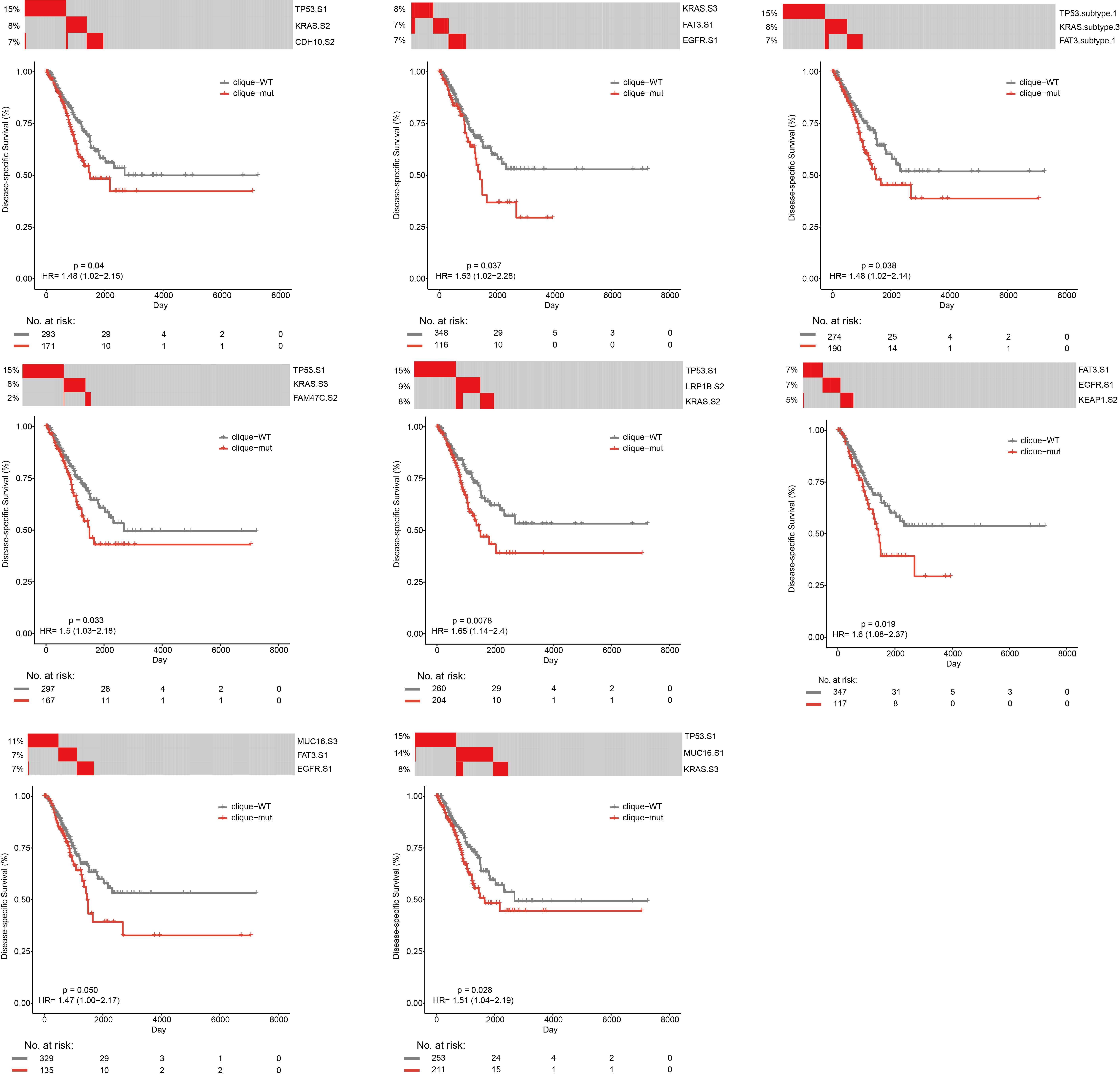


**Fig. S4** Comparison of disease-specific survival among patients carrying mutually exclusive triples (red line) and cases harboring unmutated genes (gray line) by Kaplan–Meier analysis (with logrank P values) in the cohort of LUAD patients from TCGA.

**Supplementary Tables**

**Table S1**. The table of mutually exclusive events shared by TP53 subtype one and TP53 subtype two.

| **Component1** | **Component2** | **P-value** | **Q-value** |
| --- | --- | --- | --- |
| SETD2.subtype.1 | TP53.subtype.1 | 0.0007 | 0.0011 |
| SETD2.subtype.1 | TP53.subtype.2 | 0.0185 | 0.0318 |
| KEAP1.subtype.1 | TP53.subtype.1 | 0.0014 | 0.0021 |
| KEAP1.subtype.1 | TP53.subtype.2 | 0.0174 | 0.0303 |
| KRAS.subtype.2 | TP53.subtype.1 | 0.0034 | 0.0054 |
| KRAS.subtype.2 | TP53.subtype.2 | 0.0069 | 0.0114 |
| ATM.subtype.3 | TP53.subtype.1 | 0.0063 | 0.0107 |
| ATM.subtype.3 | TP53.subtype.2 | 0.0047 | 0.0077 |
| KRAS.subtype.3 | TP53.subtype.1 | 0.0276 | 0.0494 |
| KRAS.subtype.3 | TP53.subtype.2 | <0.0001 | <0.0001 |

**Table S2**. The tables of mutually exclusive triples among driver gene mutations within subtypes.

| **Triples** | **Component1** | **Component2** | **Component3** |
| --- | --- | --- | --- |
| Triples1 | LRP1B.subtype.1 | KRAS.subtype.1 | EGFR.subtype.2 |
| Triples2 | TP53.subtype.1 | KRAS.subtype.1 | EGFR.subtype.2 |
| Triples3 | LRP1B.subtype.1 | KRAS.subtype.1 | KEAP1.subtype.13 |
| Triples4 | TP53.subtype.1 | KRAS.subtype.1 | KEAP1.subtype.1 |
| Triples5 | TP53.subtype.2 | KEAP1.subtype.1 | TLR4.subtype.1 |
| Triples6 | TP53.subtype.2 | LRP1B.subtype.1 | KEAP1.subtype.1 |
| Triples7 | LRP1B.subtype.1 | KRAS.subtype.2 | CDH10.subtype.2 |
| Triples8 | TP53.subtype.1 | KRAS.subtype.2 | CDH10.subtype.2 |
| Triples9 | TP53.subtype.2 | LRP1B.subtype.1 | KRAS.subtype.2 |
| Triples10 | TP53.subtype.1 | LRP1B.subtype.2 | KRAS.subtype.2 |
| Triples11 | CSMD3.subtype.4 | MUC16.subtype.2 | STK11.subtype.2 |
| Triples12 | KRAS.subtype.1 | EGFR.subtype.2 | APOB.subtype.1 |
| Triples13 | LRP1B.subtype.2 | EGFR.subtype.2 | APOB.subtype.1 |
| Triples14 | KRAS.subtype.1 | KEAP1.subtype.1 | CTNNA2.subtype.2 |
| Triples15 | TP53.subtype.2 | KEAP1.subtype.1 | CTNNA2.subtype.2 |
| Triples16 | KRAS.subtype.1 | EGFR.subtype.2 | CDH10.subtype.1 |
| Triples17 | LRP1B.subtype.2 | EGFR.subtype.2 | CDH10.subtype.1 |
| Triples18 | EGFR.subtype.2 | CDH10.subtype.1 | CSMD3.subtype.3 |
| Triples19 | MUC16.subtype.1 | CDH10.subtype.1 | CSMD3.subtype.3 |
| Triples20 | CSMD3.subtype.2 | KRAS.subtype.2 | CDH10.subtype.2 |
| Triples21 | LRP1B.subtype.2 | CSMD3.subtype.2 | KRAS.subtype.2 |
| Triples22 | TP53.subtype.2 | CSMD3.subtype.2 | KRAS.subtype.2 |
| Triples23 | KRAS.subtype.1 | CSMD3.subtype.2 | EGFR.subtype.2 |
| Triples24 | LRP1B.subtype.2 | CSMD3.subtype.2 | EGFR.subtype.2 |
| Triples25 | MUC16.subtype.2 | CSMD3.subtype.2 | EGFR.subtype.2 |
| Triples26 | KRAS.subtype.1 | FAT4.subtype.1 | EGFR.subtype.2 |
| Triples27 | LRP1B.subtype.2 | FAT4.subtype.1 | EGFR.subtype.2 |
| Triples28 | KRAS.subtype.1 | CSMD3.subtype.1 | KEAP1.subtype.1 |
| Triples29 | TP53.subtype.2 | CSMD3.subtype.1 | KEAP1.subtype.1 |
| Triples30 | KRAS.subtype.1 | CSMD3.subtype.1 | EGFR.subtype.2 |
| Triples31 | LRP1B.subtype.2 | CSMD3.subtype.1 | EGFR.subtype.2 |
| Triples32 | LRP1B.subtype.2 | PTPRD.subtype.2 | KRAS.subtype.2 |
| Triples33 | TP53.subtype.2 | PTPRD.subtype.2 | KRAS.subtype.2 |
| Triples34 | PTPRD.subtype.2 | KRAS.subtype.1 | KEAP1.subtype.1 |
| Triples35 | TP53.subtype.2 | PTPRD.subtype.2 | KEAP1.subtype.1 |
| Triples36 | PTPRD.subtype.2 | KRAS.subtype.1 | EGFR.subtype.2 |
| Triples37 | LRP1B.subtype.2 | PTPRD.subtype.2 | EGFR.subtype.2 |
| Triples38 | KRAS.subtype.3 | EGFR.subtype.2 | CSMD3.subtype.3 |
| Triples39 | KRAS.subtype.3 | MUC16.subtype.2 | EGFR.subtype.2 |
| Triples40 | TP53.subtype.1 | KRAS.subtype.3 | NF1.subtype.2 |
| Triples41 | TP53.subtype.1 | KRAS.subtype.3 | FAT4.subtype.2 |
| Triples42 | TP53.subtype.1 | KRAS.subtype.3 | FAM47C.subtype.2 |
| Triples43 | TP53.subtype.1 | KRAS.subtype.3 | CDH10.subtype.2 |
| Triples44 | TP53.subtype.1 | KRAS.subtype.3 | KMT2C.subtype.1 |
| Triples45 | KRAS.subtype.3 | FAT3.subtype.1 | EGFR.subtype.1 |
| Triples46 | TP53.subtype.1 | KRAS.subtype.3 | FAT3.subtype.1 |
| Triples47 | KRAS.subtype.3 | CSMD3.subtype.4 | MUC16.subtype.2 |
| Triples48 | TP53.subtype.1 | KRAS.subtype.3 | CSMD3.subtype.4 |
| Triples49 | MUC16.subtype.1 | KRAS.subtype.3 | CSMD3.subtype.3 |
| Triples50 | TP53.subtype.1 | MUC16.subtype.1 | KRAS.subtype.3 |
| Triples51 | SPTA1.subtype.1 | KRAS.subtype.3 | EGFR.subtype.1 |
| Triples52 | TP53.subtype.1 | SPTA1.subtype.1 | KRAS.subtype.3 |
| Triples53 | FAT3.subtype.2 | KRAS.subtype.2 | CDH10.subtype.2 |
| Triples54 | LRP1B.subtype.2 | FAT3.subtype.2 | KRAS.subtype.2 |
| Triples55 | TP53.subtype.2 | FAT3.subtype.2 | KRAS.subtype.2 |
| Triples56 | FAT3.subtype.2 | KRAS.subtype.1 | KEAP1.subtype.1 |
| Triples57 | TP53.subtype.2 | FAT3.subtype.2 | KEAP1.subtype.1 |
| Triples58 | FAT3.subtype.2 | KRAS.subtype.1 | EGFR.subtype.2 |
| Triples59 | LRP1B.subtype.2 | FAT3.subtype.2 | EGFR.subtype.2 |
| Triples60 | KEAP1.subtype.2 | KRAS.subtype.1 | EGFR.subtype.2 |
| Triples61 | LRP1B.subtype.2 | KEAP1.subtype.2 | EGFR.subtype.2 |
| Triples62 | KEAP1.subtype.2 | FAT3.subtype.1 | EGFR.subtype.1 |
| Triples63 | KEAP1.subtype.2 | KRAS.subtype.1 | EGFR.subtype.1 |
| Triples64 | SPTA1.subtype.1 | KEAP1.subtype.2 | EGFR.subtype.1 |
| Triples65 | MUC16.subtype.3 | KEAP1.subtype.1 | CSMD3.subtype.3 |
| Triples66 | TP53.subtype.2 | MUC16.subtype.3 | KEAP1.subtype.1 |
| Triples67 | MUC16.subtype.3 | KRAS.subtype.1 | EGFR.subtype.2 |
| Triples68 | MUC16.subtype.3 | LRP1B.subtype.2 | EGFR.subtype.2 |
| Triples69 | MUC16.subtype.3 | EGFR.subtype.2 | CSMD3.subtype.3 |
| Triples70 | MUC16.subtype.3 | FAT3.subtype.1 | EGFR.subtype.1 |
| Triples71 | MUC16.subtype.3 | SPTA1.subtype.1 | EGFR.subtype.1 |
| Triples72 | SPTA1.subtype.2 | KRAS.subtype.2 | CDH10.subtype.2 |
| Triples73 | SPTA1.subtype.2 | LRP1B.subtype.2 | KRAS.subtype.2 |
| Triples74 | TP53.subtype.2 | SPTA1.subtype.2 | KRAS.subtype.2 |
| Triples75 | SPTA1.subtype.2 | KRAS.subtype.1 | KEAP1.subtype.1 |
| Triples76 | TP53.subtype.2 | SPTA1.subtype.2 | KEAP1.subtype.1 |
| Triples77 | SPTA1.subtype.2 | KRAS.subtype.1 | EGFR.subtype.2 |
| Triples78 | SPTA1.subtype.2 | LRP1B.subtype.2 | EGFR.subtype.2 |
| Triples79 | TP53.subtype.2 | SPTA1.subtype.2 | SETD2.subtype.1 |

**Table S3** Multivariate analysis for a mutually exclusive triple of disease-specific survival in the cohort (TP53.subtype.1.MUC16.subtype.1.KRAS.subtype.3).

| Variables |  |  | Univariate |  |  |  | Multivariate |  |
| --- | --- | --- | --- | --- | --- | --- | --- | --- |
|  |  | HR | 95% CI | p value |  | HR | 95% CI | p value |
| Age |  | 0.990 | 0.972-1.009 | 0.315 |  | 0.996 | 0.977-1.016 | 0.696 |
| AJCC stage | stage II vs I | 3.439 | 2.067-5.719 | <0.001* |  | 1.682 | 0.462-6.127 | 0.431 |
|  | stage III vs I | 3.072 | 1.936-4.873 | <0.001* |  | 1.490 | 0.671-3.305 | 0.327 |
|  | stage IV vs I | 4.311 | 2.068-8.987 | <0.001* |  | 3.155 | 1.328-7.495 | 0.009* |
| T stage | T2 vs T1 | 1.574 | 0.998-2.484 | 0.051 |  | 1.325 | 0.816-2.149 | 0.255 |
|  | T3 vs T1 | 2.922 | 1.491-5.725 | 0.002* |  | 2.150 | 0.936-4.938 | 0.071 |
|  | T4 vs T1 | 2.619 | 1.004-6.832 | 0.049* |  | 1.726 | 0.567-5.254 | 0.337 |
| N stage | N1 vs N0 | 2.773 | 1.799-4.274 | <0.001* |  | 1.834 | 0.881-3.819 | 0.105 |
|  | N2 vs N0 | 2.888 | 1.761-4.738 | <0.001* |  | 1.566 | 0.481-5.088 | 0.455 |
|  | N3 vs N0 | 3.936 | 0-Inf | 0.995 |  | 1.431 | 0-Inf | 0.994 |
| Gender | Male vs Female | 0.943 | 0.645-1.379 | 0.762 |  | 0.787 | 0.525-1.182 | 0.249 |
| TP53.subtype.1.MUC16.subtype.1.KRAS.subtype.3 | Mutations vs wildtype | 1.476 | 1.010-2.155 | 0.044* |  | 1.475 | 0.986-2.207 | 0.048* |

Significant *P* values are labeled with * (*P* < 0.05).
